# Difficulty with the preceding visual search affects brain activity in the following resting period

**DOI:** 10.1038/s41598-022-21624-3

**Published:** 2022-11-03

**Authors:** Ayumi Takemoto, Sunao Iwaki, Zhoumao Duo, Shinobu Yasumuro, Takatsune Kumada

**Affiliations:** 1 Vision Sensing Lab., Technology Research Center, Technology and Intellectual Property H.Q., OMRON Corporation, Kyoto, Japan; 2 Human Informatics and Interaction Research Institute, National Institute of Advanced Industrial Science and Technology (AIST), Tsukuba, Japan; 3 Graduate School of Comprehensive Human Sciences, University of Tsukuba, Tsukuba, Japan; 4 Graduate School of Informatics, Kyoto University, Kyoto, Japan; 5 Present Address: Faculty of Computing, University of Latvia, Riga, Latvia; 6 Present Address: School of Integrative and Global Majors, University of Tsukuba, Tsukuba, Japan; 7 Bioinformatics Laboratory, Riga Stradins University, Riga, Latvia

**Keywords:** Attention, Perception

## Abstract

It has been well-documented that brain regions related to a task are activated during the task performance. We investigated whether brain activity and functional connectivity during the rest period are affected by the preceding task. Participants performed visual search tasks with three search conditions, which were followed by a rest period. During the rest period, participants were asked to look at the display that did not show any visual stimuli. In the result, brain activity in occipital and superior parietal regions would be deactivated by the preceding task during the rest period after visual search tasks. However, the activity of the inferior frontal gyrus during the rest period, which is also part of the attention network, was not affected by the brain activity during the preceding visual search task. We proposed a new model for explaining how the cognitive demands of the preceding visual search task regulate the attention network during the rest period after the task. In this model, the cognitive demand changes with task difficulty, which affects the brain activity even after removing the visual search task in the rest phase.

## Introduction

There is ample behavioral evidence that the current cognitive processing affects the following processing. Such psychological phenomena were found in a wide variety of psychological processing, from lower sensory-processing level (e.g., sensory adaptation^1,2^, or visual masking^3,4^) to motor-execution level (e.g., psychological refractory period^5^). A typical phenomenon is “priming”, in which the preceding cognitive processing of stimuli positively or negatively affects processing the following stimuli^6^. For example, in repetition priming experiments, preceding presentation of a stimulus facilitates the processing of the same stimulus^7^. The repetition priming was also found in visual search experiments. When several types of a target were presented in a task and the same type of a target was presented in consecutive trials, the detection of the second target was facilitated compare to when a different type of a target was presented in the preceding trial^8,9^. On the other hand, the negative effect of the preceding presentation of stimuli, referred to as the negative priming, was also reported. Typically, performance to an object which is ignored in the preceding presentation decreased relative to a trial in which the object was not presented in the preceding presentation^10^. From the physiological point of view, some traces of brain activities in regions activated in the previous processing may persist into the timing of the following stimulus presentation, affecting the processing of the stimuli. Most brain imaging studies investigating priming effect focused on the response of brain regions to repeated stimuli. A mechanism of priming in the brain called "repetition suppression (RS)" was proposed^11^, in which, when the same stimulus is repeatedly presented, the brain activity for the stimulus decreases. RS is also considered a type of adaptation and has been found in various regions of the brain such as left fusiform gyrus, right/left extrastriate cortex and left precentral sulcus^12^. In these previous studies of aftereffect, the aftereffects of preceding visual tasks were examined on the basis of the response to the following stimuli.

In this study, we focused on the brain state after the termination of a visual task. We assume that brain regions engaged in the preceding task may show traces of a preceding visual task and show activity ready for the next task. However, most research focused on brain activity in many types of tasks, including visual search tasks, and the brain activity when presenting blank displays (without targets or distractors) or fixation displays (a plus sign in the display) is generally used as the baseline. Thus, to the best of our knowledge, there have been no studies that have reported brain activities during the rest period after tasks. We investigated the effect of previous visual search tasks on brain activity during the resting period after the tasks. The reason we used a visual search task is as follows. Since such tasks have been widely used in psychophysical and brain-imaging studies^13,14^, the brain mechanism involved in the task has been widely documented. Second, the task difficulty or degree of involvement of the top-down attentional control can be easily manipulated by varying the relationship between a target and distractors. When a target is defined by the difference in a single visual feature (such as color or orientation) from distractors, the target can be found with relatively short reaction times (RTs), and the RTs are almost independent from the number of distractors on the display. This type of search is referred to as efficient search^15^. When a target is defined by the combination of visual features or when a target is quite similar to the distractors, RTs increase as a function of the number of distractors on the display. This type of search is referred to as inefficient search. Different brain regions are activated in efficient search than in inefficient search. For example, Nobre et al. (2003) reported that the occipital and parietal regions are more active in inefficient tasks than in efficient tasks. Wei et al. (2009) reported that the activity of the superior frontal gyrus (SFG) for inefficient visual search display was lower than for efficient visual search display, which was regarded as the deactivation of the right temporoparietal junction (TPJ), and the role of the right SFG in filtering out and rejecting distracting information from clutter items^16^. These regions are parts of the attention network, which includes occipital gyrus (OG), inferior parietal sulcus (IPS), TPJ, and SFG of the brain^17^.

We assumed that the effect on the brain activity during a rest period depends on the brain activity in the previous visual -search-task, specifically, the part of the brain that is more active in a visual-search-task has more of an effect on the brain activity during rest period after task, and less active during visual search task has less effects on the brain activity during the rest period after the visual search task. Thus, we expected that efficient and inefficient tasks would affect the activity of regions belonging to the attention network differently and these effect would induce brain activity in the rest periods after tasks. If we find brain activity during a rest period, we should determine whether the activity has the same functional role as the attentional network driven by visual stimuli input as during a visual search task or has different functions. Furthermore, Maximo et al. (2016) reported that functional connectivity in the attention network responds to task difficulty in visual tasks^18^. Thus, we analyzed psychophysiological interactions (PPIs) using the CONN toolbox^19^ to investigate the effect of the preceding task difficulty on the interactions between OG and other brain regions in the resting period. PPI analysis estimates task-specific changes in the relationship between regions of interests (ROIs) and brain regions^20^. The right OG region, which is a part of the primary visual regions of the brain and the inputting point of visual stimuli, was selected as a seed ROI in our PPI analysis,because attention network is dominant in the right hemisphere^17^. We considered the relationship between the right OG and other areas related to attention network. We assumed that the connectivity among brain regions which involves the attention network in a visual search task, engaged the brain attention network afterward during the rest period, even though searching displays have been deleted. A non-search task was also presented as the baseline condition on similar displays to the search task for clarifying the saccade effects on the brain activity during the rest period after the task.

## Results

First, a post-hoc analysis was conducted by G$*$Power^21^ to confirm sufficient statistical power (Power = .996). We next compared the percentage of questions answered correctly. There was a significant difference between Diff-Task (mean = 73.30, standard deviations (SD) = 14.59) and Easy-Task (mean = 96.02, SD = 6.00) (*t*(21) = 7.00, $\textit{p} < 0.01$). The brain activities while participants were looking for targets were focused on in this research, thus, all trials were included in subsequent analyses. We compared the *Task-phase* and *Blank-phase* data under (1) *(Non)* and *(Easy/Diff)* experimental conditions and (2) inefficient *(Diff)*/efficient *(Easy)* visual-search-task conditions.

### Comparison of brain activation between task difficulties in *Task-phase* and *Blank-phase*

**Figure 1:**
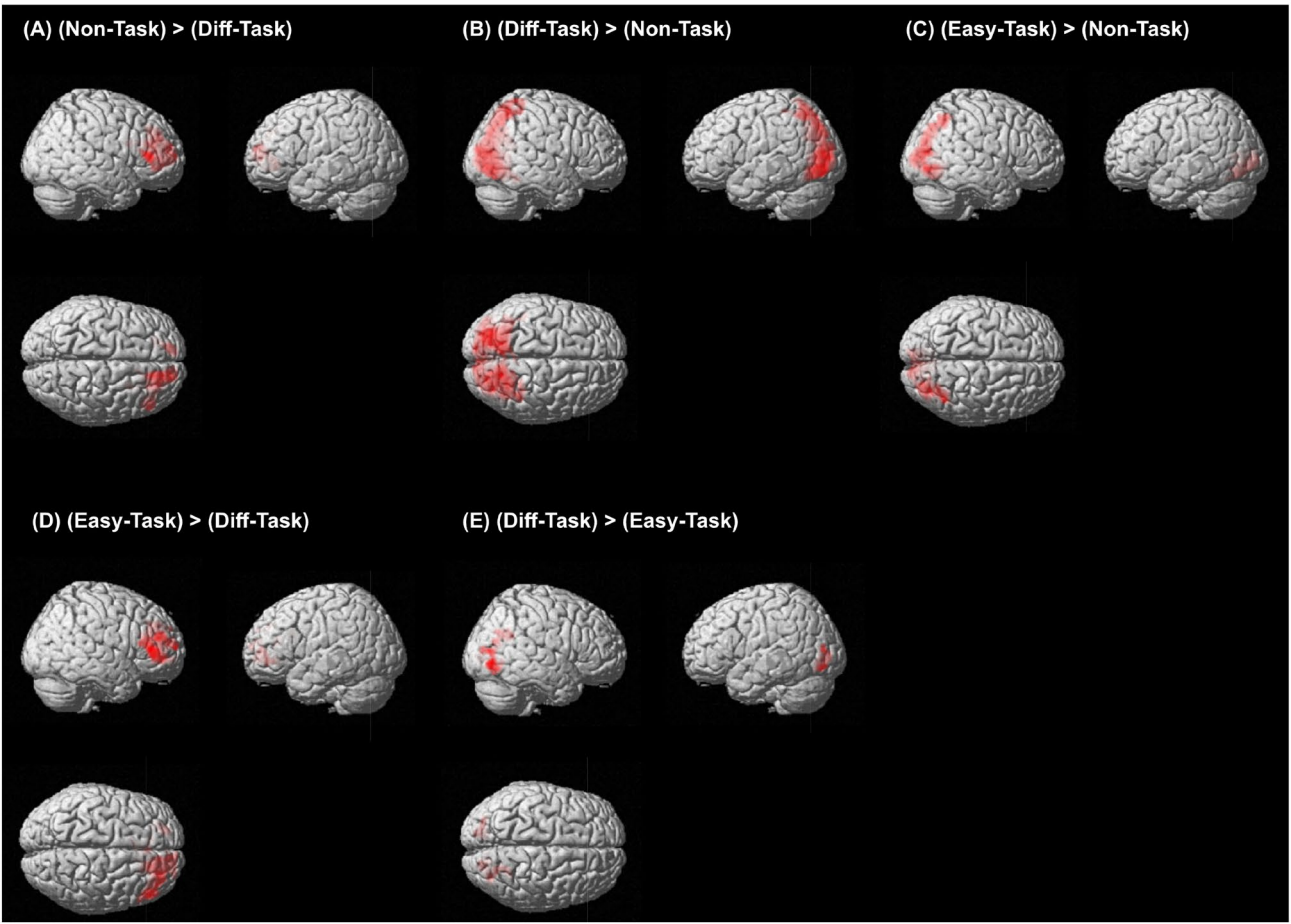
Areas that were more activated in *Task-phase* (voxel level threshold of $p_{uncorr} < 0.001$ with cluster level threshold of $p_{FWE-corrected} < 0.05$). (**A**) BOLD signals in these areas were higher in *Non-Task* than in *Diff-Task*, (**B**) in *Diff-Task* than in *Non-Task*, (**C**) in *Easy-Task* than in *Non-Task*, (**D**) in *Easy-Task* than in textitDiff-Task, and (**E**) in *Diff-Task* than in *Easy-Task*.

We separately analyzed BOLD signals in the *Task-phase* and the *Blank-phase*. We first compared the brain activity in *Diff-Task* versus *Non-Task* and *Easy-Task* versus *Non-Task* to clarify the effect of task demand on visual search. The right middle frontal gyrus (MFG), SFG, inferior frontal gyrus (IFG), and anterior cingulate cortex (ACC) were more active in *Non-Task* than in *Diff-Task* (Fig. 1A and Supplementary Table S1). In contrast, the OG and lingual gyrus were more active in *Diff-Task* than in *Non-Task* (Fig. 1B and Supplementary Table S1). In *Easy-Task*, the OG and lingual gyrus were more active than in *Non-Task* (Fig. 1C and Supplementary Table S1). These regions were similar in *Diff-Task*, but there were no more activated regions in *Non-Task* than in *Easy-Task*.

We then investigated the differences in the brain activities between task difficulties. In *Easy-Task*, the IFG, MFG, SFG, ACC, and right frontal operculum were more activated than in *Diff-Task* (Fig. 1D and Supplementary Table S2). Furthermore, the OG, lingual gyrus, and the right superior parietal lobe (SPL) were more active in the *Diff-Task* than in *Easy-Task* (Fig. 1E and Supplementary Table S2).

**Figure 2:**
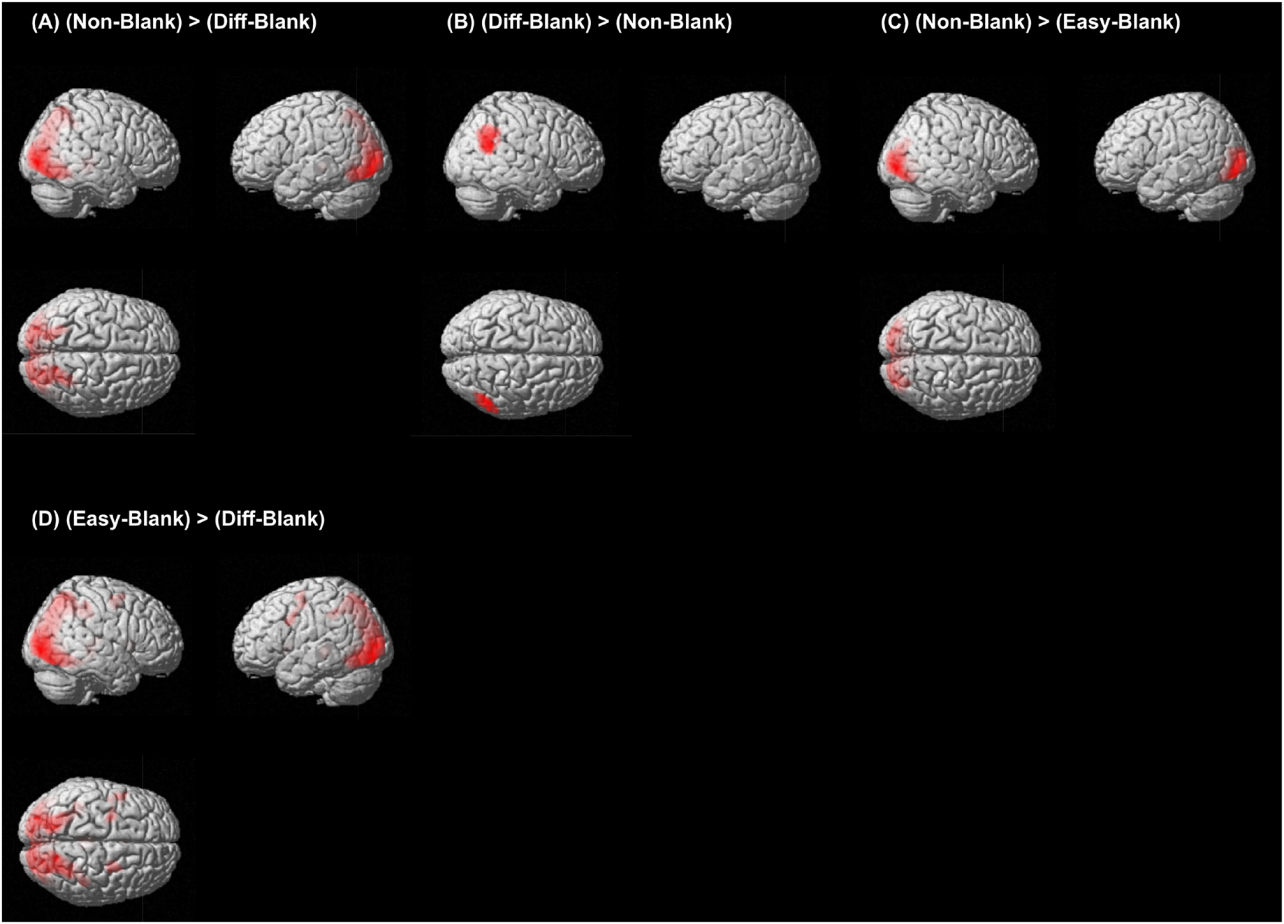
Areas were more activated in *Blank-phase* (voxel level threshold of $p_{uncorr} < 0.001$ with cluster level threshold of $p_{FWE-corrected} < 0.05$). (**A**) BOLD signals in these areas were higher in *Non-Blank* than in *Diff-Blank*, (**B**) in *Diff-Blank* than in *Non-Blank*, (**C**) in *Non-Blank* than in *Easy-Blank*, and (**D**) in *Easy-Blank* than in *Diff-Blank*.

Next, we compared the effect of the presence of targets on brain activity in *Blank-phase*; *Diff-Blank* versus *Non-Blank* and *Easy-Blank* versus *Non-Blank*. The results indicate that the right angular gyrus was more active in *Diff-Blank* than in *Non-Blank* (Fig. 2B and Supplementary Table S3), but the OG and left lingual gyrus were more active in *Non-Blank* than in *Easy- and Diff-Blank* (Fig. 2A,C and Supplementary Table S3). No regions were more active in *Easy-Blank* than in *Non-Blank*.

We examined the differences between the task difficulties’ effect on the brain in the *Blank-phase*. The OG, SFG, MFG, posterior cingulate cortex(PCC), thalamus, and right ACC were more active in *Easy-Blank* than in *Diff-Blank* (Fig. 2D and Supplementary Table S4). However, no regions were more active in *Diff-Blank* than in *Easy-Blank*.

### Comparison of beta values between task difficulties in *Task-phase* and *Blank-phase*

**Figure 3:**
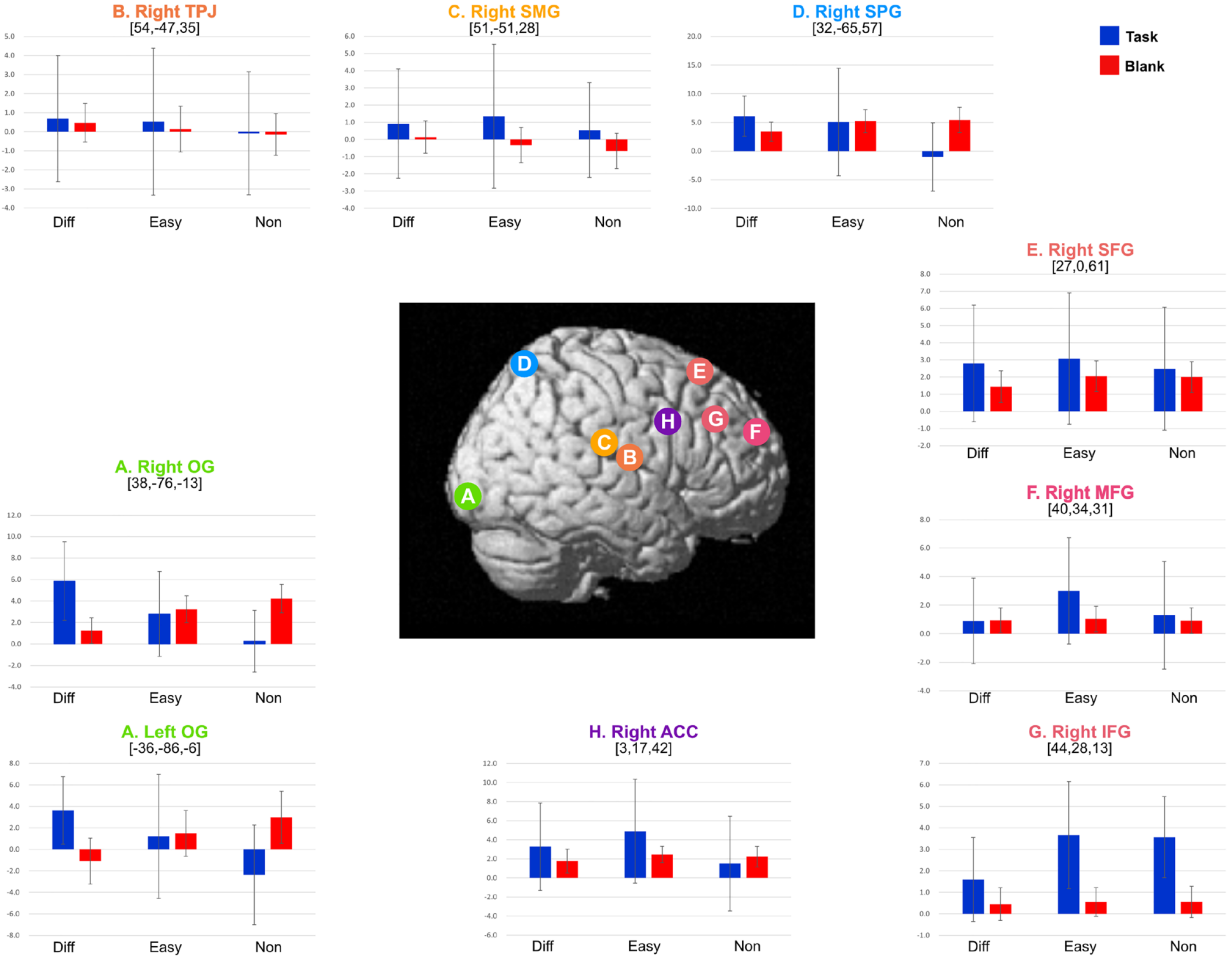
Beta values of all ROIs in all phases. Illustration of brain in middle indicates position of each ROI. (**A**) Right/left OG; occipital gyrus, (**B**) right TPJ; temporoparietal junction, (**C**) right SMG; supramarginal gyrus, (**D**) right SPG; superior parietal gyrus, (**E**) right SFG; superior frontal gyrus, (**F**) right MFG; middle frontal gyrus, (**G**) right IFG; inferior frontal gyrus, and (**H**) right ACC; anterior cingulate gyrus. The bar graphs indicate the average, and the error bars are standard deviations.

We calculated the beta values of nine ROIs related to visual attention network and the default mode network (DMN). These included the OG and right TPJ/supramarginal gyrus (SMG)/superior parietal gyrus (SPG)/SFG/MFG/IFG/ACC. We conducted a one-way analysis of variance with the task as the main factor then calculated the Bonferroni adjusted p-value for multiple tests. The results are shown in Fig. 3 and Supplementary Table S5. There were four types of beta value patterns that matched task difficulties and phases. The beta values of the OG and right SPG for the *Task-phase* and the *Blank-phase* were affected by task difficulties.Supplementary Table S5 shows that the beta value of the OG in the *Task-phase* increased in the order of *Non-Task, Easy-Task,* and *Diff-Task* (the blue bars of Fig. 3A). However, in the *Blank-phase*, the beta values decreased in the order of *Non-Blank, Easy-Blank,* and *Diff-Blank* (the red bars of Fig. 3A). The right SPG’s beta value was the lowest for the three search conditions in *Non-Task* (the blue bar of Fig. 3D). However, in the *Blank-phase*, the beta value in *Diff-Blank* was lower than in the others (the red bars of Fig. 3D). The beta value in the *Task phase* were affected by task difficulties. Specifically, the activities of the right IFG in *Task-phase* were different between task difficulties. The beta value of the right IFG in *Diff-Task* was lower than those of the other regions (the blue bars of Fig. 3G). However, there were no significant changes in the *Blank-phase* (the red bars of Fig. 3G). The beta value in the *Blank-phase* were affected by task difficulties, but there were no changes in the right SMG in the *Task-phase*. The beta value in the right SMG increased in the order of *Non-Blank, Easy-Blank,* and *Diff-Blank* (the red bars of Fig. 3C). Finally, the beta values of the right TPJ, right SFG, right MFG, and right ACC did not show significant changes in either the *Task-phase* or *Blank-phase* (Fig. 3B,E,F,H).

### Effect of task difficulties on brain network in *Task-phase* and *Blank-phase*

**Figure 4:**
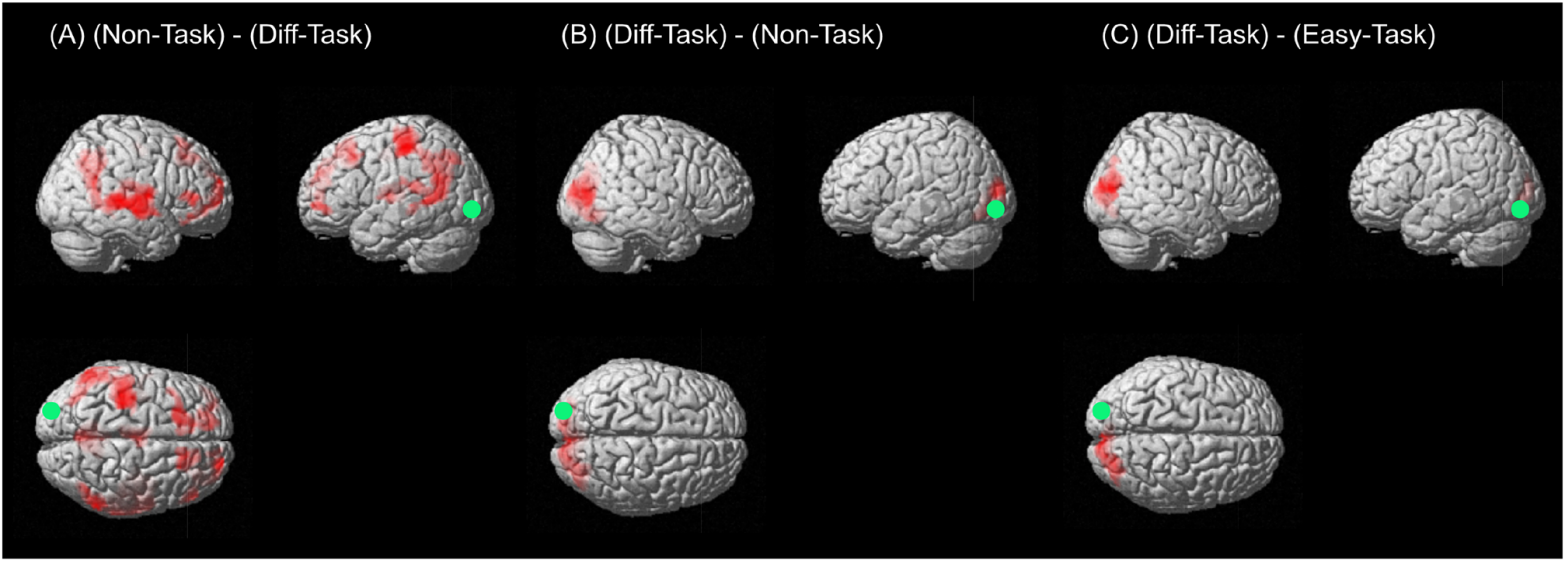
PPI results with right OG as seed (voxel level threshold of $p_{uncorr} < 0.001$ with cluster level threshold of $p_{FWE-corrected} < 0.05$). (**A**) Areas are more closely related to right OG in *Non-Task* than in *Diff-Task*. (**B**) in *Diff-Task* than in *Non-Task* and (**C**) in *Diff-Task* than in *Easy-Task*. Green circles indicate the seed in each figure but actual size was sphere diameter of 6 mm.

We investigated the brain network by PPI in the *Task-phase* and textitBlank-phases using the right OG as a seed because it has been reported that the OG is engaged in a variety of visual tasks including attention, visual search, and eye movement^22,23^. We focused on the visual-search-task demands and difficulties. We first compared *Non* with *Easy and Diff*. The results indicate that the right/left lingual gyrus, right/left cuneus, and right/left OG were more highly correlated with the right OG in *Diff-Task* than in *Non-Task* (Fig. 4B, Supplementary Table S6). The left postcentral gyrus, left SPG, left central operculum, SFG, MFG, precentral gyrus, supplementary motor cortex, precuneus, cuneus, SMG, and right angular gyrus were more closely related to the right OG in *Non-Task* than in *Diff-task* (Fig. 4A, Supplementary Table S7).

We then compared task difficulties. The cuneus, OG, and lingual gyrus in the *Task-phase* were more closely related to the right OG in *Diff-Task* than in *Easy-Task* (Fig. 4C and Supplementary Table S6).

There was no regions closely related to the right OG in the *Blank-phase*.

## Discussion

Most studies have reported on brain activity during visual tasks.To the best of our knowledge, this is the first study to examine brain activity after performing a task. Brain activity and connectivity during a rest period are affected by the mental demand of preceding visual search task. On the basis of our results, we developed a model to explain how top-down and bottom-up task demands during preceding task periods modulate the brain network in the following rest period. We examined brain activation and connectivity in the *Blank-phase* during which no task was performed, after conducting visual search tasks with various task demand levels during the *Task-phase*. The results indicate that the brain activity during the *Blank-phase* was modulated by the difficulty of the preceding visual search task. The significance of these findings is discussed in more detail in the following subsections.

### Brain activity and connectivity in the *Task-phase*

The difficulty of a visual search task in the *Task-phase* affected the brain activity related to the attention network. The right OG indicated higher functional connectivity to the attention network in *Diff-Task* than in *Easy-Task* and *Non-Task*. The attention network was more closely related to the DMN in *Non-Task* than *Diff-Task*.

The bilateral OG, lingual gyrus, and right SPG were more active when performing more difficult tasks (Figs. 1 and 3). These results are consistent with previous studies involving visual search tasks. For example, inefficient conjunction search tasks require deeper visual processing and dorsal and ventral stream activities depending on task difficulty^24^. Nobre et al. (2003) reported that the occipital regions and superior/inferior parietal regions are activated in different visual search tasks that included efficient features, inefficient features, efficient conjunctions and inefficient conjunctions. The right OG and SPL regions specifically indicated activation during inefficient search tasks compared with saccade tasks^25^. Therefore, performing *Diff-Task* that required more overt and covert attentional control than *Easy-* or *Non-Task* would be expected to induce higher activity in the posterior regions of the frontoparietal attention network.

The SFG, MFG, and IFG were more active in the easier task trials than in more difficult task trials (see *Non-Task* versus *Diff-Task* and *Easy-Task* versus *Diff-Task* ) (Figs. 1 and 3, and Supplementary Table S1 and S2). One study indicated that the magnitude of the right SFG activities in visual search for heterogeneous displays (conjunction visual search) was lower than for homogeneous displays (feature search), which was interpreted as due to the deactivation of the TPJ and the crucial role of the right SFG in filtering out and rejecting distracting information from clutter items^16^. It was also reported that the right MFG and IFG is connected to the right TPJ^23^ that the right SFG, right TPJ, MFG, IFG and precuneus are deactivated during visual search tasks, and that the deactivation of frontal areas and the precuneus reflects the filtering of irrelevant inputs from the TPJ, preventing unimportant objects from being attended^14,26^. Therefore, a filter might determine the range of stimuli inputted to the TPJ, and the dorsal frontoparietal regions may be involved in setting up this filter^14,26^. Our results support these studies. The BOLD signals in the right SFG and MFG were stronger in the relatively easier task phases (Fig. 1). Furthermore, the magnitude of activity in the right IFG had a higher degree of deactivation in *Diff-Task* (Fig. 3). We suggest that the SFG, MFG, and IFG had a higher degree of deactivation in *Diff-Task* because the conjunction of features defined a target in *Diff-Task* and these brain regions were deactivated by filtering the target from the distractor.

The study also indicated that task difficulty affected the connectivity between specific brain regions and the right OG in the *Task-phase*. The bilateral lingual gyri and cuneus were more closely related to the OG in *Diff-Task* than in *Easy-Task* and *Non-Task*. These aregions represent the primary visual cortex, which engages visual processing of primitive visual features such as motion, contrast changes, and spatial frequency^27^. Parker et al. (2014) reported that the bilateral cuneus showed high activation in conjunctive visual search tasks, followed by the lingual gyrus and middle OG. Vabbu et al. (2001) reported that the OG and cuneus is simultaneously activated by visual stimuli. Maximo et al. (2016) showed that dorsal visual stream areas were more strongly related to the lingual gyrus, occipital pole, precentral gyrus, and SMG during difficult visual search tasks than easy tasks because more brain resources are needed for optimal goal-driven visual search^18^. Our results are consistent with those results of these studies. Considering the findings of these studies, we suggest that the connectivity between the right OG and other occipital regions was more robust in *Diff-Task* than *Easy-Task* or the *Non-Task* because *Diff-Task* required the highest level of attentional and visual processing to find a target. In *Non-Task*, however, the right OG was more closely related to the SMG, SFG, and MFG which are parts of the TPJ. The right TPJ positively correlated with the DMN and deactivated when searching through displays containing distractors compared with the baseline^26^. The connectivity between the right OG and right TPJ in *Non-Task* was more robust than in *Diff-Task* which was also consistent with the past study^26^. We suggest that the right TPJ was more closely related to the right OG in *Non-Task* because the DMN engaged in *Non-Task*, and positively correlated with the right TPJ.

### Brain activity and connectivity in *Blank-phase*

The difficulty in the *Task-phase* significantly affected the brain activity in the *Blank-phase*.

The magnitude of the activity in the bilateral OG, right SPG, and SMG depended on the difficulty of the *Task-phase*. The bilateral SPG and OG were activated in *Diff-Task* more than in *Easy-Task* and *Non-Task*. However, they had higher degree of deactivation in *Diff-Blank* (Figs. 2 and 3, and Supplementary Table S5) The bilateral OG and right SPG are known to be active during inefficient visual search tasks^25^. *Diff-Task* required a higher level of visual processing because it was a conjunction search task^24^. We assumed that these regions were resting in the *Blank-phase*, and the resting level would depend on the difficulty of the preceding tasks.

The right SMG in *Diff-Blank* was more highly active than in *Easy-Blank* and *Non-Blank* (Figs. 2 and 3, and Supplementary Table S5). The right SMG which is part of DMN, is known to be deactivated by goal-driven attentional control, and activated by the stimuli-driven control of attention^28^. It is also activated when people make self-referential decisions focusing on internal information concerning their current mental states^29^. We suggest that the DMN would be more active after performing the *Diff-Task* than *Easy-Task* and *Non-Task* for resting the visual regions by focusing on the internal environment during the rest period^30,31^.

We also found that task difficulties did not affect the connectivity between specific brain regions and the right OG in the *Blank-phases*, although the brain activity was affected by task difficulty. This indicates that the aftereffect of the *Task-phase* would reflect each brain region related to the attention network directly in the *Blank-phase*. Thus, the OG and SPL, which are parts of the attention network, were affected by task difficulty not only in the *Task-phase* but also in the *Blank-phase*, although there was no connectivity between the right OG and brain regions of the attention network.

### Model of brain activity in task and rest periods

**Figure 5:**
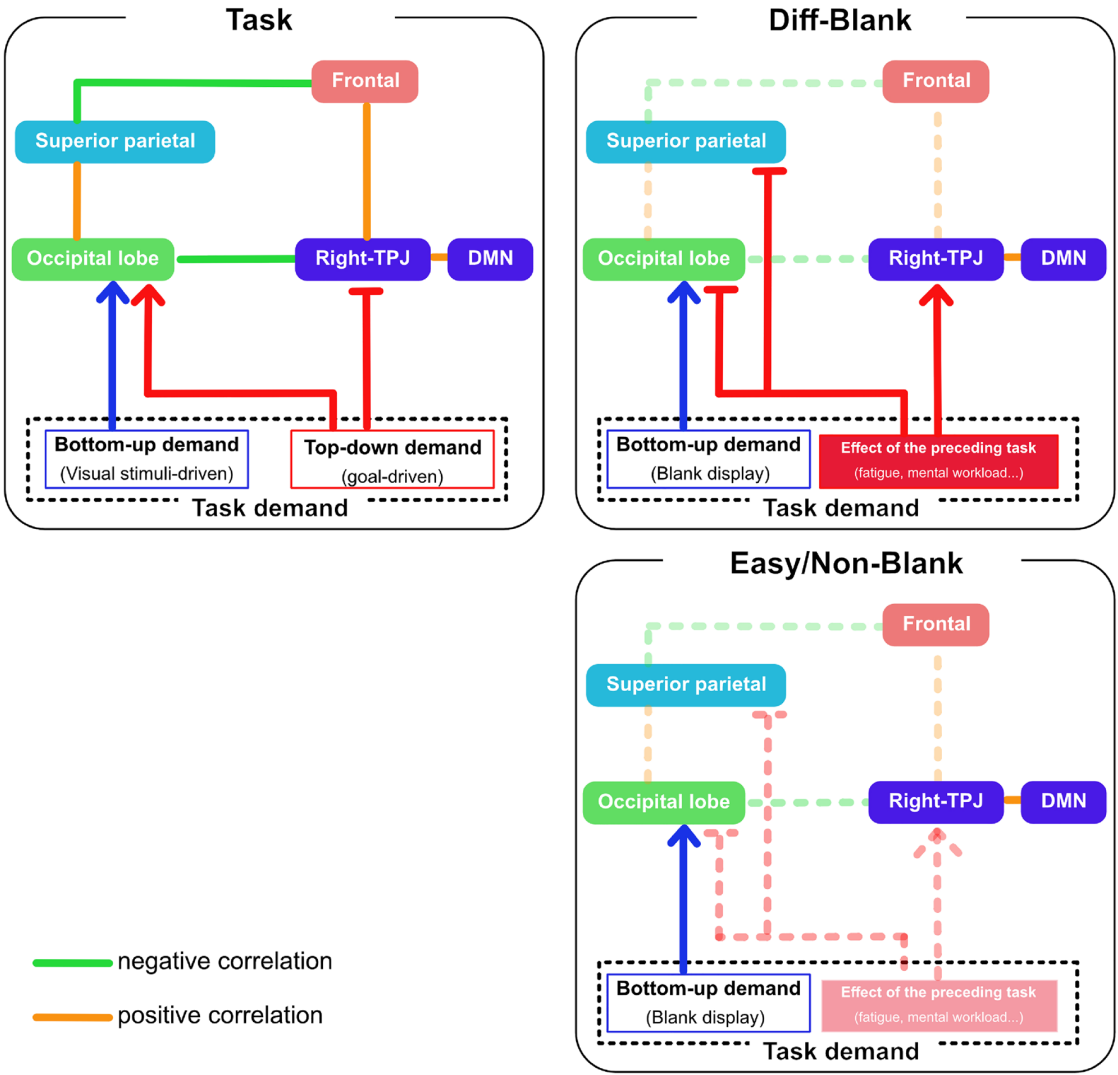
Model of the brain network considering difficulty during task and blank phases. Green lines indicate negative correlations, and orange lines indicate positive correlations. Blue arrows indicate bottom-up demand and red arrows indicate effect of top-down demands in task phase and the aftereffects of top-down demands during a rest period after task phase. Left figure is the model of the brain network during task. Right figures indicate the model of the brain network during a rest period after task. Lower figure shows the network after easy task, and upper right figure shows the network after difficult task during a rest period.

We developed a model for explaining how brain activities in blank phases reflected brain activities in task phases, on the basis of past studies^14,22,25,26^ and the results of this study. Shomstein (2012) summarized the scheme of the attentional network which consists of the occipital lobe, TPJ, SPL, and IFG. Both SPL and TPJ induce signals responsible for subsequent attentional modulations observed in the occipital lobe^22^. Furthermore, enhanced activation of OG and SPL reflects increased demands during visual search, namely, these areas are more activated in inefficient search tasks than in simple saccade tasks^25^. We naturally assumed that the bottom-up (visual stimulus-driven) and top-down (goal-driven) demands would activate the visual cortex, mainly the occipital lobe in task phases. A positive connection between the occipital lobe and dorsal attention network (mainly the SPG) was demonstrated. Therefore, these regions would also be activated by top-down control and bottom-up control of visual processing. However, these controls deactivate the right TPJ, and the right TPJ has positive connections with the DMN and IFG^14,26^. As a result, the IFG would also be deactivated by the top-down and bottom-up controls (left figure in Fig. 5). The bottom-up physical demands did not vary between task difficulties because the visual displays consisted of 50 items irrespective of the tasks. The effect of top-down demand differed between task difficulties. Therefore, the activity of these regions depends on the goal-driven demand resulting from task difficulty.

Our results suggest that the aftereffect was led by the preceding visual tasks during the rest period after a visual search task, although the bottom-up demands were constant for participants, irrespective of task difficulty because they looked at the same blank display without any distractors or targets (right figures in Fig. 5). However, the magnitude of the aftereffect depended on the difficulty of the preceding visual search task. As a result, the right TPJ and DMN engaged proactively, and the occipital lobe and SPG deactivated because the visual processing system had a higher tendency to rest after difficult tasks than easy tasks during a rest periods.

## Methods

### Participants

Healthy participants (N = 33) were recruited for this study. The study was approved by the ethics committee of the National Institute of Advanced Industrial Science and Technology in accordance with the Declaration of Helsinki (approval number: 2017-740). All participants provided written informed consent before the experiment. The data of 11 participants who did not answer the thought-probe more than 5 $\%$ or displays excessive head movement in the magnetic resonance imaging (MRI) scanner, defined as translational or rotational displacements greater than 3.0mm in any direction, were excluded from this study. Therefore, the data of 22 participants were analyzed (ratio of right-handers to left-handers = 21:1, age range 22.00±3.22, 11 men and 11 women).

### Apparatus

Structural and functional data were obtained using a 3-Tesla MRI scanner (Philips Ingenia 3.0T; Philips, Netherlands) with a 32-channel head coil located at the National Institute of Advanced Industrial Science and Technology (AIST). The visual stimuli were presented on an MR-compatible LCD display (32" NNL LCD Monitor; NordicNeuroLab AS, Norway) placed at the end of the scanner bore, 174 cm from the participants’ eyes, and spanning a visual angle of 22 degrees horizontally and 13 degrees vertically. The participants viewed the display through a mirror attached to the head coil. The behavioral responses were collected using an MR-compatible fiber-optic button box (HHSC-1x5-D; Current Designs Inc., USA). The stimulus presentation and response collection was controlled using PsychoPy^32^, a software library written in Python for Intel-based PCs.

### Procedure

**Figure 6:**
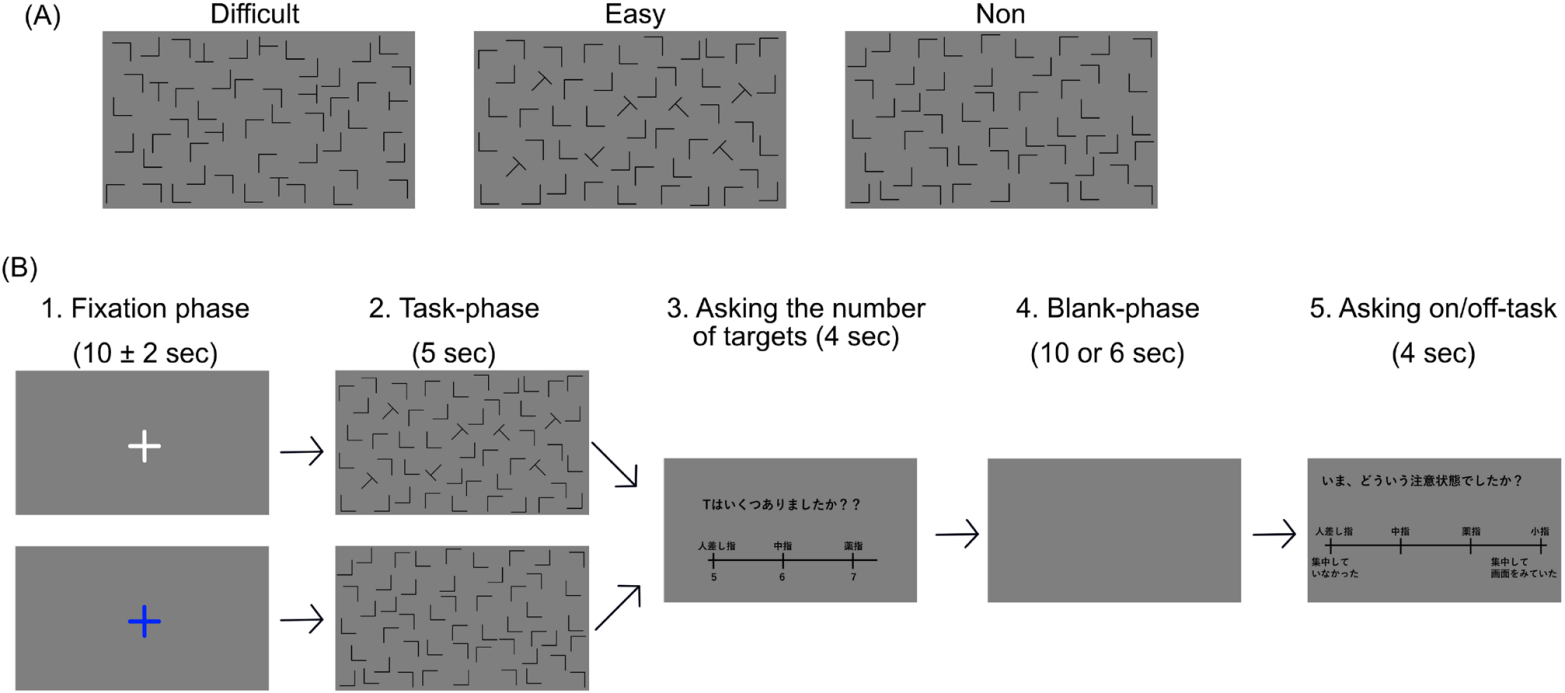
(**A**) Visual stimuli examples and (**B**) experimental procedure. 1. *fixation phase* in which white plus informed participants of next trial as being either *Diff* or *Easy*. Blue plus informed participants that next trial would be *Non*., 2. *Task phase*, 3. display asked participants "How many ’T’s were there in previous display ?" (in Japanese), and participants answered by pressing button., 4. *Blank phase*, and 5. display asking "Did you focus on the task ?" (in Japanese), and participants answered by pressing button.

Figure 6B illustrates the experimental procedure. Participants took part in four sessions with a five-minute rest period between sessions. Each session lasted approximately 15 min and consisted of 25 trials (9 trials of *Diff*, 9 trials of *Easy*, and 7 trials of *Non*) which were presented in a random order, namely each participant completed 100 trials.

Figure  1 shows the task sequence of each trial. In the first phase, *’Fixation’* phase, a fixation display was presented for 10 ± 2 s while the participants looked at a blue or white plus sign at the center of the display (Fig. 6B-1). The white plus sign informed the participants that the next display would present either a *Diff* or *Easy* trial. The blue plus sign informed them that the next trial would be a *Non* trial. In the *Task-phase*, a visual search display was presented for 5 s, which allowed the participants to count the number of targets (Fig. 6B-2). After *Diff and Easy-Task* phases, a question display was randomly presented 4 times in each session for 4 s, which showed a question in Japanese: "How many ’T’s are there in the display?". Participants were asked to report the number of targets by pressing a bottom. The correct ratio was calculated by counting the number of the correct answers to questions. A blank screen was presented in the *Blank-phase* for either 6 or 10 s while the participants were looking at the display (Figure 6B-3), which was presented for 6 s after the question display had been presented. After these phases, a thought-probe showing the sentence (in Japanese), "Did you focus on the task" was displayed for every trial (Fig. 6B-4), however, such information is not presented in this paper. Participants reported whether they had concentrated by pressing one of four corresponding keys. The display returned to the fixation display after this phase. When planning this study, we were also interested in the mind-wandering state of participants during the rest period. Thus, we presented a thought-probe display in every trial. However, since mind-wandering reports were largely sporadic, only a small number of participants were suitable for the analysis involving the mind-wandering state as an independent variable. Therefore, we do not report on the results related to the mind-wandering state in this paper. Some participants did not respond to certain some thought-probes. Thus, we assumed they were not fully awake during those trials

### Stimuli

Figure 6A shows examples of the visual search displays. We used ’L’s as distractors and ’T’s as targets. They were presented in black against a gray background at a height and width of approximately 3.5 cm. We used three task conditions: difficult *(’Diff’)*, in which the targets rotated ± 90 degrees; (2) easy *(’Easy’)*, in which the targets rotated ± 45 and ± 135 degrees; and (3) no targets *(’Non’)*. There were three combinations of target and distractor in *Diff* and *Easy* trials, such that a display consisted of 43 distractors and 7 targets, 44 distractors and 6 targets, or 45 distractors and 5 targets and a display consisted of 50 distractors in *Non* trials. A gray background without any targets or distractors was presented in the blank display.

### fMRI data acquisition

Each participant’s head was fixed using foam padding to reduce head movements. Single-shot echo-planar imaging (EPI) sequences were used to acquire functional images. The EPI parameters were as follows: repetition time (TR) = 2000 ms; echo time (TE) = 35 ms; flip angle = 90-degrees, 31 ascending slices, thickness = 3.7 mm.

### Image processing and statistical analysis

#### fMRI activation analysis

The SPM12 software (https://www.fil.ion.ucl.ac.uk/spm/software/spm12/) on Matlab 2020a was used for MRI data analysis. All coordinates are reported in standard Montreal Neurological Institute (MNI) space. We used a general linear model (GLM) for first-level event-related analysis of each participant. In the GLM analysis, trials of the experiment were modelled as box-car functions representing cue stimuli, task and blank onsets and durations for each condition convolved with a hemodynamic response function. Task state events (*’Diff-Task’, ’Easy-Task’, ’Non-Task’*) were modeled as a duration of 5 s from the onset of the search display convolved with the hemodynamics response function, and Blank state events (*’Diff-Blank’, ’Easy-Blank’, ’Non-Blank’*) were modeled as a duration of 10 s from the onset of the blank display convolved with the hemodynamics response function. The Blank state events after participants were asked the number of targets, were excluded from the analysis to avoid the side-effects of answering the questions. We performed whole-brain analysis for every contrast between conditions. A second-level analysis used contrasts from the first-level and one-sample tests to investigate group-level activation. The initial uncorrected voxel threshold was set to $p < 0.001$. Clusters were considered as significant if they fell below a cluster-corrected *p*(FWE) = 0.05. Beta values were extracted from the ROIs using Marsbar (http://marsbar.sourceforge.net/)^33,34^. These values were used to generate plots representing the beta values of the participants in the contrast of interest. In the beta value analysis, we defined MNI coordinates of ROIs that satisfied the following criteria; (1) the spherical ROI coordinates should show significant activation (*p* < 0.001) in a cluster-based FWE in the second-level SPM analysis; and (2) past studies have reported that the regions are involved in visual attention network. The maximum difference between (1) and (2) was within 31 mm along any axes of the MNI coordinate. To investigate the effect of the preceding visual search task on the brain activity during the rest period after the task, we selected these ROIs and computed the difference in beta values between the *fixation phase* and each experimental condition (*Diff-Task, Easy-Task, or Non-Task* and *Diff-Blank, Easy-Blank, or Non-Blank*). We selected 9 regions for calculating the beta values on the basis of these criteria: right/left OG (Right: Brodmann area (BA) 37, x = 38 , y = − 76 , z = − 13, Left: BA 18, x = − 36, y = – 86, z = − 6), right IFG (BA 46, x = 44, y = 28, z = 13), the right SPL (BA 7, x = 32, y = − 65, z = 57)^25^, right MFG (BA 9, x = 40, y = 34, z = 31)^14^, right TPJ (BA 39, x = 54, y = − 47, z = 35)^35^, right SMG (BA 39, x = 51, y = -51, z = 28)^14^, SFG (BA 6, x = 27, y = 0, z = 61)^36^, and ACC (BA 8, x = 3, y = 17, z = 42)^37^.

#### Psychophysiological interactional analysis

Psychophysiological interactions (PPIs) using the CONN toolbox^19,20^ was conducted to understand the effect of the connection of the brain activity in each phase. We chose the ROIs of the right OG (BA 37, x = 38, y = – 76, z = − 12, sphere diameter of 6 mm) as the seed that engaged with the visual pathway, as previously reported^25^. PPI analysis involves using psychological, physiological and interactional regressors. The entire blood oxygenation level dependent (BOLD) time series in the right OG was used as the physiological and psychological regressors, which are interesting contrasts of the experimental conditions (*Task: Diff-Task versus Non-Task, Diff-Task versus Easy-Task, Blank: Non-Blank versus Diff-Blank, Easy-Blank versus Diff-Blank*). The interactional regressors, which are the product of physiological and psychological regressors, were generated separately for each combination of experimental conditions. A second-level analysis involved contrasts from the first level and one-sample tests to investigate the group-level effects of the preceding visual search tasks. The initial uncorrected voxel threshold was set to $p < 0.001$. Clusters below a cluster-corrected *p*(FWE) of 0.05 were considered significant.

## Supplementary Information

Supplementary Information.

## Data Availability

The datasets from the current study are available from the corresponding author on reasonable request.
