# Supplementary material for: Difficulty with the preceding visual search affects brain activity in the following resting period
Source: Sci Rep. 2022 Nov 3;12:18545. doi: 10.1038/s41598-022-21624-3 (PMC9633596; doi:10.1038/s41598-022-21624-3)
Supplement: Supplementary file 1 — Supplementary Information. [file 41598_2022_21624_MOESM1_ESM.pdf]

Visual-search-task-difficulty affects brain activity in following rest period

Ayumi Takemoto, Sunao Iwaki, Zhoumao Duo, Shinobu Yasumuro, and Takatsune Kumada

Supplemental Table S1. Comparison of BOLD signal in *Non-Task* vs *Diff-Task* or *Easy-Task*

	#cluster	Regions	x	y	z	peak z state	cluster size	p
**(Non-Task) - (Diff-Task)**	1	R-superior frontal gyrus<br>R-anterior cingulate gyrus<br>R-middle frontal gysur	18	38	24	5.10	2455	<<0.001
		R-superior frontal gyrus<br>R-frontal pole<br>R-middle frontal gyrus	16	56	8	5.06		
		R-inferior frontal gyrus<br>R-frontal operculum<br>R-middle frontal gyrus	48	30	8	4.76		
	2	L-superior frontal gyrus<br>L-anterior cingulate gyrus	-14	52	16	4.28	429	0.006
		L-superior frontal gyrus<br>L-frontal pole<br>R-superior frontal gyrus	-8	60	18	4.01		
		L-inferior frontal gyrus<br>L-anterior orbital gyrus	-24	38	0	3.91		
**(Diff-Task) - (Non-Task)**	1	L-lingual gyrus<br>L-occipital fusiform gyrus<br>L-calcarine cortex	-14	-82	-8	6.55	15622	<<0.001
		L-calcarine cortex<br>L-lingual gyrus<br>L-cuneus<br>L-occipital pole	-8	-88	0	5.94		
		R-calcarine cortex<br>R-lingual gyrus<br>L-lingual gyrus<br>R-cuneus<br>L-calcarine cortex<br>L-cuneus<br>R-occipital pole	8	-88	-2	5.76		
**(Easy-Task) - (Non-Task)**	1	L-calcarine cortex<br>L-lingual gyrus<br>L-cuneus<br>L-occipital pole<br>R-lingual gyrus	-6	-90	-2	5.29	4750	<<0.001
		R-calcarine cortex<br>R-lingual gyrus<br>R-occipital pole<br>L-calcarine cortex<br>L-lingual gyrus<br>L-cuneus<br>R-cuneus	6	-92	-2	4.98		
		R-lingual gyrus<br>R-occipital fusiform gyrus<br>R-fusiform gyrus	20	-66	-6	4.98		

Supplemental Table S2. Comparison of BOLD signal in Easy-*Task* vs *Diff-Task*

	#cluster	Regions	x	y	z	peak z state	cluster size	p
**(Easy-Task) - (Diff-Task)**	1	R-middle frontal gyrus<br>R-superior frontal gyrus<br>R-anterior orbital gyrus	24	48	0	5.32	2917	<<0.001
		R-inferior frotnal gyrus<br>R-middle frontal gyrus<br>R-frontal operculum	44	34	8	5.30		
		R-inferior frotnal gyrus<br>R-middle frontal gyrus<br>R-frontal operculum	44	38	0	4.60		
	2	R-anterior cingulate gyrus<br>L-anterior cingulate gyrus<br>R-middle cingulate gyrus<br>R-superior frontal gyrus<br>L-middle cingulate gyrus	2	32	18	3.91	259	0.034
		L-middle cingulate gyrus<br>R-middle cingulate gyrus<br>L-anterior cingulate gyrus<br>L-supplementary motor cortex<br>R-anterior cingulate gyrus<br>L-superior frontal gyrus	-4	18	28	3.60		
		R-anterior cingulate gyrus<br>L-anterior cingulate gyrus<br>R-middle cingulate gyrus<br>L-middle cingulate gyrus	4	22	14	3.53		
	3	L-superior frontal gyrus<br>L-middle frontal gyrus<br>L-anterior cingulate gyrus<br>L-anterior orbital gyrus<br>L-medial frontal cortex	-18	50	-2	3.79	322	0.015
		L-anterior orbital cortex<br>L-posterior orbital cortex<br>L-inferior frontal gyrus<br>L-medial orbital cortex<br>L-lateral obital cortex<br>L-anterior insula	-24	36	-4	3.68		
		L-superior frontal gyrus<br>L-middle frontal gyrus<br>L-anterior cingulate gyrus	-18	52	6	3.63		
**(Diff-Task) - (Easy-Task)**	1	R-occipital fusiform gyrus<br>R-lingual gyrus	24	-74	-10	4.44	1030	<<0.001
		R-precuneus<br>R-cuneus<br>R-superior parietal lobule<br>R-superior occipital gyrus<br>R-angular gyrus<br>R-middle occipital gyrus	24	-62	26	4.24		
		R-lingual gyrus<br>R-occipital fusiform gyrus<br>R-calcarine cortex	18	-78	-6	4.19		
	2	L-lingual gyrus<br>L-occipital fusiform gyrus	-16	-80	-10	4.21	488	0.002
		L-calcarine cortex<br>L-lingual gyrus	-14	-88	0	3.76		
		L-calcarine cortex<br>L-lingual gyrus	-10	-80	0	3.74		

Supplemental Table S3. Comparison of BOLD signal in *Non-Blank* vs *Diff-Blank or Easy-Blank*

	#cluster	Regions	x	y	z	peak z state	cluster size	p
**(Non-Blank) - (Diff- Blank)**	1	R-inferior occipital gyrus<br>R-middle occipital gyrus<br>R-occipital pole<br>R-superior occipital gyrus	32	-86	2	inf	16334	<<0.001
		L-inferior occipital gyrus<br>L-middle occipital gyrus<br>L-superior occipital gyrus<br>L-occipital pole	-28	-88	2	7.78		
		L-lingual gyrus<br>L-calcarine cortex<br>R-lingual gyrus	-8	-84	-4	7.57		
**(Diff-Blank) - (Non-Blank)**	1	R-angular gyrus<br>R-middle temporal gyrus<br>R-middle occipital gysus<br>R-inferior occipital gyrus	58	-60	20	4.90	638	0.004
		R-angular gyrus	56	-58	30	4.32		
		R-angular gyrus	48	-62	42	4.23		
**(Non-Blank) - (Easy-Blank)**	1	R-lingual gyrus<br>R-calcarine cortex<br>L-lingual gyrus<br>R-cuneus	10	-86	-4	6.59	6437	<<0.001
		L-calcarine cortex<br>L-lingual gyrus	-10	-86	-2	6.59		
		R-occipital fusiform gyrus<br>R-inferior occipital gyrus<br>R-lingual gyrus	28	-82	-4	6.51		

Supplemental Table S4. Comparison of BOLD signal in *Easy-Blank* vs *Diff-Blank*

	#cluster	Regions	x	y	z	peak z state	cluster size	p
**(Easy-Blank) - (Diff-Blank)**	1	L-inferior occipital gyrus<br>L-middle occipital gyrus<br>L-superior occipital gyrus	-26	-86	4	7.43	17545	<<0.001
		R-occipital fusiform gyrus<br>R-inferior occipital gyrus<br>R-lingual gyrus	26	-86	-6	7.43		
		L-lingual gyrus<br>L-calcarine cortex<br>L-occipital fusiform gyrus	-10	-84	-6	7.33		
	2	L-thalamus proper<br>L-hippocampus<br>L-parahippocampal gyrus<br>L-posterior cingulate gyrus	-20	-32	0	6.08	430	0.008
	3	R-thalamus proper<br>R-hippocampus<br>R-parahippocampal gyrus<br>R-posterior cingulate gyrus	20	-30	0	5.71	661	0.001
	4	R-precentral gyrus<br>R-middle frontal gyrus<br>R-superior frontal gyrus	28	-6	52	4.61	331	0.022
		R-middle frontal gyrus<br>R-superior frontal gyrus<br>R-precentral gyrus	30	6	60	3.66		
	5	L-precentral gyrus<br>L-middle frontal gyrus<br>L-superior frontal gyrus	-30	-4	62	4.59	503	0.004
		L-precentral gyrus<br>L-superior frontal gyrus<br>L-middle frontal gyrus	-24	-8	52	4.24		
		L-precentral gyrus<br>L-middle frontal gyrus	-52	4	42	4.23		
	6	R-posterior cingulate gyrus<br>L-posterior cingulate gyrus<br>R-middle cingulate gyrus	8	-36	26	4.55	375	0.014
		L-posterior cingulate gyrus<br>R-posterior cingulate gyrus<br>L-middle cingulate gyrus	-4	-38	24	3.87		
		R-posterior cingulate gyrus<br>R-precuneus	10	-42	14	3.29		
	5	L-anterior cingulate gyrus<br>R-anterior cingulate gyrus	-4	14	-2	3.63	411	0.010

Supplemental Table S5. Beta values in *Task-phase* and *Blank-phase*

ROIs	[x,y,z]		beta-value			p-value
			Diff	Easy	Non	
Right occipital	[38,-76,-13]	Task	5.85± 3.65	2.80± 3.96	0.26± 2.87	<0.01
		Blank	1.25± 1.20	3.23± 1.26	4.22± 1.32	<0.01
Left occipital	[-36,-86,-6]	Task	3.62± 3.14	1.21± 5.78	-2.37± 4.63	<0.01
		Blank	-1.08± 2.14	1.48± 2.13	2.98± 2.42	<0.01
Right TPJ	[54,-47,35]	Task	0.68± 3.31	0.53± 3.86	-0.08± 3.23	1.00
		Blank	0.47± 1.02	0.14± 1.20	-0.14± 1.10	0.17
Right SMG	[51,-51,28]	Task	0.92± 3.18	1.35± 4.19	0.54± 2.75	1.00
		Blank	0.13± 0.94	-0.32± 1.03	-0.66± 1.02	0.02
Right SPG	[32,-65,57]	Task	6.08± 3.50	5.07± 9.36	-1.03± 6.00	<0.01
		Blank	3.39± 1.66	5.22± 2.01	5.43± 2.20	<0.01
Right SFG	[27,0,61]	Task	2.79± 3.40	3.08± 3.83	2.48± 3.58	1.00
		Blank	1.44± 0.92	2.05± 0.90	2.00± 0.90	0.11
Right MFG	[40,34,31]	Task	0.90± 3.00	3.00± 3.71	1.29± 3.77	0.71
		Blank	0.94± 0.86	1.03± 0.89	0.92± 0.89	1.00
Right IFG	[44,28,13]	Task	1.60± 1.95	3.66± 2.49	3.56± 1.89	<0.01
		Blank	0.45± 0.76	0.55± 0.67	0.55± 0.73	1.00
Right ACC	[3,17,42]	Task	3.28± 4.58	4.90± 5.45	1.51± 4.97	1.00
		Blank	1.77± 1.25	2.46± 0.85	2.22± 1.08	1.00

p-value is Bonferroni adjustment 18*p-value

Supplemental Table S6. PPI results that more highly correlated with the right OG in *Diff-Task* than in *Non-Task* or *Easy-Task*

	#cluster	Regions	x	y	z	peak z state	cluster size	p
(Diff-Task) - (Non-Task)	1	R-calcarine cortex<br>R-lingual gyrus<br>R-occipital fusiform gyrus	20	-78	0	4.96	4328	<<0.001
		R-inferior occipital gyrus<br>R-middle occipital gyrus	42	-80	4	4.92		
		R-calcarine cortex<br>R-lingual gyrus<br>L-lingual gyrus<br>R-cuneus<br>L-calcarine cortex<br>L-cuneus	6	-82	2	4.79		
(Diff-Task) - (Easy-Task)	1	R-cuneus<br>L-cuneus<br>R-occipital pole<br>R-calcarine cortex<br>L-occipitla pole<br>R-superior occipital gyrus	6	-92	18	4.06	1509	<<0.001
		R-lingual gyrus<br>L-lingual gyrus	8	-80	-12	3.96		
		R-superior occipital gyrus<br>R-occipitla pole<br>R-cuneus	20	-90	20	3.85		

Supplemental Table S7. PPI results that more highly correlated with the right OG in *Non-Task* than in *Diff-Task*

	#cluster	Regions	x	y	z	peak z state	cluster size	p
**(Non-Task) - (Diff-Task)**	1	L-precentral gyrus<br>L-postcentral gyrus	-38	-20	62	5.33	2284	<<0.001
		L-postcentral gyrus<br>L-supramarginal gyrus<br>L-superior parietal lobule	-50	-28	54	4.93		
		L-postcentral gyrus<br>L-precentral gyrus<br>L-superior parietal lobule	-34	-32	68	4.62		
	2	R-angular gyrus<br>R-supramarginal gyrus	60	-50	34	5.23	3739	<<0.001
		R-superior temporal gyrus<br>R-middle temporal gyrus	66	-32	4	5.18		
		R-angular gyrus	46	-62	44	5.10		
	3	L-superior frontal gyrus<br>L-middle frontal gyrus	-20	22	38	4.64	1687	<<0.001
		L-superior frontal gyrus<br>L-middle frontal gyrus	-24	22	50	4.28		
		L-superior frontal gyrus	-14	52	28	4.00		
	4	L-precuneus<br>R-precuneus	-6	-64	36	4.59	1409	<<0.001
		R-precuneus<br>R-posterior cingulate gyrus	10	-52	36	4.55		
		L-precuneus<br>L-cuneus<br>R-precuneus<br>L-calcarine cortex<br>R-cuneus<br>L-posterior cingulate gyrus<br>R-calcarine cortex<br>R-lingual gyrus<br>L-lingual gyrus	-4	-62	18	3.65		
	5	L-supplementary motor cortex<br>L-middle cingulate gyrus<br>R-supplementary motor cortex<br>R-middle cingulate gyrus<br>L-precentral gyrus<br>R-precentral gyrus	-4	-10	48	4.24	513	0.009
		L-middle cingulate gyrus<br>L-precentral gyrus<br>L-supplementary motor cortex<br>R-middle cingulate gyrus<br>R-precentral gyrus<br>R-supplementary motor cortex<br>L-posterior cingulate gyrus	-4	-18	46	4.20		
		R-supplementary motor cortex<br>R-precentral gyrus<br>L-supplementary motor cortex<br>L-precentral gyrus<br>R-superior frontal gyrus	6	-12	62	3.12		
	6	R-fontal pole<br>R-superior frontal gyrus<br>R-middle frontal gyrus<br>R-anterior orbital gyrus	20	60	0	4.23	1123	<<0.001
		R-inferior frontal gyrus<br>R-middle frontal gyrus<br>R-frontal operculum	48	36	2	4.12		
		R-inferior frontal gyrus<br>R-frontal operculum	56	32	6	4.10		

	#cluster	Regions	x	y	z	peak z state	cluster size	p
**(Non-Task) - (Diff-Task)**	7	R-superior frontal gyrus<br>R-middle frontal gyrus	22	24	44	4.16	477	0.012
		R-superior frontal gyrus<br>R-supplementary motor cortex	16	26	56	3.73		
		R-superior frontal gyrus<br>R-supplementary motor cortex	10	22	62	3.67		
	8	L-central operculum<br>L-posterior insula<br>L-transverse temporal gyrus<br>L-parietal operculum<br>L-anterior insula	-36	-16	16	3.78	622	0.004
		L-central operculum<br>L-posterior insula<br>L-anterior insula	-36	-8	14	3.61		
		L-transverse temporal gyrus<br>L-posterior insula<br>L-parietal operculum<br>L-central operculum<br>L-planum polare<br>L-planum temporale	-36	-24	10	3.59		
